# Factors associated with reproductive health and health education participation among female college students in China

**DOI:** 10.3389/fpubh.2025.1627669

**Published:** 2025-09-01

**Authors:** Yiyi Zhuang, Kim Kwang Cheol, Mary Jane Botabara-Yap, Kuan Zhao, Rowena Imelda A. Ramos, Wenming Cao

**Affiliations:** ^1^Health Industry Department, College of Health Industry, Xiamen Donghai Vocational and Technical College, Xiamen, Fujian, China; ^2^Yonsei University, Seoul, Republic of Korea; ^3^Adventist University of the Philippines, Cavite, Philippines; ^4^The Clinical Nutrition Department, Qingdao Public Health Clinical Center, Qingdao, China; ^5^Department of Gynecology, Pingshan District Central Hospital, Shenzhen, China

**Keywords:** reproductive health, health education, HPV vaccine hesitancy, sociodemographic determinants, Chinese female college students

## Abstract

**Purpose:**

To investigate sociodemographic determinants of reproductive health disparities and health education participation among Chinese female college students (CFCs).

**Methods:**

A nationally representative sample of 1,013 students from 12 provinces (October to November 2024) completed validated questionnaires. Multilevel logistic regression analyzed clustered data (school-level ICC = 0.19).

**Results:**

Significant associations were observed between sociodemographic factors education level, household registration, only child status, academic major and reproductive health outcomes (*p* < 0.05). Key findings include pronounced urban–rural inequities, with urban students demonstrating 4.3-fold higher HPV vaccination rates than rural peers (78.5% vs. 45.7%, aOR = 4.3, 95% CI: 3.2–5.8), alongside elevated dysmenorrhea prevalence among rural students (56.9% vs. 43.5%, aOR = 1.8, 95% CI: 1.4–2.3). Academic stressors significantly impacted health outcomes, as postgraduate students exhibited a 60% higher dysmenorrhea risk versus undergraduates (60.9% vs. 50.8%, aOR = 1.6, 95% CI, 1.2–2.1), while paradoxically, medical students showed lower HPV vaccination uptake than non-medical peers (58.0% vs. 74.3%, aOR = 2.1), attributed to clinical skepticism about vaccine safety. Furthermore, health education engagement was limited (46.1% participation), with 52.4% relying on online platforms for health information—highlighting critical gaps in institutional health promotion and digital misinformation risks. Therefore, addressing these multifaceted socioeconomic, educational, and structural barriers is essential for improving reproductive health equity in this population.

**Conclusion:**

Multifaceted strategies addressing socioeconomic barriers, health education gaps, and digital misinformation are critical to improving reproductive health in female college students.

## 1 Introduction

Reproductive health remains a cornerstone of global health equity, particularly among young women in transitional societies (1). In China, female college students face compounded challenges: 52% report menstrual disorders (2), and human papillomavirus (HPV) vaccination rates (62%) lag developed Asian peers (2). These issues impose substantial economic burdens (3). Despite growing recognition, gaps persist in understanding context-specific determinants of health behaviors (4).

In China, female college students face compounded challenges rooted in its unique sociocultural context. The urban–rural divide in healthcare resources, intensified by the legacy of the *hukou* system, exacerbates disparities in reproductive health access. China’s hukou system perpetuates urban–rural healthcare disparities, with rural students facing 50% reduced gynecological care access (2). Additionally, the postgraduate entrance exam craze (a unique stressor for 78% of Chinese undergraduates) correlates with elevated cortisol levels and menstrual dysregulation (5). This study uniquely integrates the Health Belief Model (HBM) to dissect how perceived barriers (e.g., vaccine cost) and self-efficacy (e.g., medical literacy) interact with structural inequities in shaping health behaviors.”

Female college students represent a vulnerable demographic at a critical developmental stage, facing unique biopsychosocial challenges. The physical and psychological stages are mature but not yet sound. The reproductive health problems of female college students are often easily overlooked. Currently, the understanding of reproductive system diseases is still insufficient, and more in-depth research is urgently needed to fill this gap. A review of the literature at home and abroad revealed relatively few studies on the reproductive health of a specific group of female college students. Female college students generally face problems such as a lack of knowledge and insufficient awareness of diseases in terms of reproductive health (6). Therefore, it is particularly important to study the reproductive health status of female college students and its influencing factors (5).

The Health Belief Model (HBM) provides a theoretical framework, positing that health behaviors stem from perceived benefits, barriers, and self-efficacy (7). Prior studies highlight the HBM’s utility in explaining vaccination hesitancy (8) and menstrual health management (9). However, its application in China’s unique sociocultural context—characterized by academic pressure gradients and urban–rural healthcare divides—remains underexplored (10).

This study addresses three objectives: Assess demographic profiles and reproductive health status of CFCs. Identify sociodemographic factors influencing dysmenorrhea, irregular menstruation, and breast disease. Evaluate health education’s role in improving reproductive health management.

## 2 Methods

### 2.1 Study design and data collection

This cross-sectional study was conducted from October 1 to November 20, 2024. The research team sent out electronic questionnaires through the online platform “Questionnaire Star” and collected data.^1^ The survey period aligned with the academic calendar to maximize participation and minimize disruptions during examinations (11, 12). The survey was administered and monitored by trained research assistants, with follow-up reminders sent weekly to non-respondents.

### 2.2 Questionnaire development and validation

The questionnaire was designed using validated scales adapted to the sociocultural context of China. It comprised three sections:

(1) Demographics: Age, education level (associate degree, undergraduate, postgraduate), household registration (urban/rural), academic major (medical/non-medical), and only-child status.

(2) Reproductive Health Status (6): Validated scales assessed dysmenorrhea (Cronbach’s = 0.79), irregular menstruation (*α* = 0.82), and breast disease (*α* = 0.81).

(3) Health Education and HPV Vaccine Hesitancy: The WHO Vaccine Hesitancy Scale was culturally adapted through forward-backward translation and pilot testing (*α* = 0.77). Health education participation was measured using a 5-point Likert scale.

The WHO scale underwent forward-backward translation by bilingual public health experts and cultural adaptation via focus group discussions (*n* = 20). Pilot testing (n = 100) informed revisions to ambiguous terms (e.g., “vaccine safety”). Exploratory factor analysis (EFA) with Promax rotation identified three latent factors: reproductive health knowledge (*α* = 0.79), healthcare access perceptions (*α* = 0.82), and digital literacy (*α* = 0.71). Confirmatory factor analysis (CFA) confirmed model fit (RMSEA = 0.06, CFI = 0.93, TLI = 0.91).

### 2.3 Participant selection and sampling

A nationally representative sample of 1,050 female college students was recruited from 12 provinces in China (Fujian, Guangdong, Sichuan, Henan, Zhejiang, Shandong, Liaoning, Yunnan, Shaanxi, Jiangsu, Hubei, and Heilongjiang), selected via stratified random sampling to reflect geographic diversity (coastal vs. inland) and urban–rural population distribution (urban: 62.9%, rural: 37.1%). The 37.1% rural sample proportion aligns with the 2023 National Census showing 36.7% of college students holding rural household registration (*χ*^2^ = 0.21, *p* = 0.646). The sample size was calculated based on a 95% confidence level (*Z* = 1.96), an expected prevalence of reproductive health issues of 50% (to maximize variability), and a margin of error of ±3%, resulting in a minimum requirement of 1,067 participants. Our final sample (*n* = 1,013) aligns closely with this target. Provinces were stratified based on the National Bureau of Statistics’ classification of socioeconomic development tiers (Tier 1 to Tier 3). This approach ensured proportional representation of China’s diverse student population, covering key demographic variables such as household registration (urban/rural), academic major (medical/non-medical), and education level. To address potential clustering effects, multilevel logistic regression was applied (school-level ICC = 0.19), which statistically adjusts for intra-cluster correlations and enhances the generalizability of findings to the broader population of CFCs. Similar sampling strategies with comparable sample sizes (*n* ≈ 1,000) have been validated in nationally representative studies on youth health behaviors (10, 13). Of 1,050 distributed questionnaires, 1,013 valid responses were retained (96.5% response rate). Participants’ mean age was 20.4 years (SD = 1.8), with balanced representation of medical (48.9%) and non-medical majors (51.1%).

Inclusion Criteria: Full-time female students aged 18–30 years. Willingness to provide informed consent. Ability to complete the questionnaire independently.

Exclusion Criteria: Withdrawal during study. Incomplete or inconsistent responses (e.g., missing data >10%).

### 2.4 Statistical analysis

Data was analyzed using IBM SPSS Statistics 22.0 integrated with R 4.2.3. Missing values (<2.1% per variable) were addressed via multiple imputation. Missing data (<2.1% per variable) were imputed using fully conditional specification (FCS) via the R `mice` package, with 10 iterations and predictive mean matching. Descriptive statistics included frequencies with Wilson score 95% confidence intervals (CIs). Group differences were assessed using Pearson’s χ^2^ tests (Yates ‘correction where appropriate) and Fisher’s exact tests. Multivariable logistic regression models adjusted for age, household registration, and academic major, with results reported as adjusted odds ratios (aORs) and 95% CIs. Intraclass correlation coefficients (ICC) confirmed significant school-level clustering (ICC = 0.19, *p* < 0.001), justifying multilevel modeling. Model adequacy was verified via Hosmer–Lemeshow goodness-of-fit tests (*p* > 0.05) and variance inflation factors <1.8. The Benjamini-Hochberg procedure controlled the false discovery rate (FDR = 0.05). Statistical significance was set at two-tailed *p* < 0.05. Missing data mechanisms were verified using Little’s MCAR test (*χ*^2^ = 12.34, *p* = 0.195), supporting the missing-at-random assumption. Multiple imputation was performed with 20 iterations using predictive mean matching.

### 2.5 Ethical considerations

Approval was obtained from the Institutional Review Boards of Xiamen Donghai Institute (No. XDHI-2024-033) and Pingshan District Central Hospital (No. PSCH-2024-112). All participants provided written informed consent, and data were anonymized.

### 2.6 Rationale for methodology

The cross-sectional design was selected to capture contemporaneous associations between sociodemographic factors and reproductive health outcomes. Stratified sampling ensured representation of China’s urban–rural divide and academic diversity. The inclusion of medical and non-medical students facilitated comparative analyses of health literacy impacts. Ethical compliance and rigorous statistical methods aligned with international standards for reproducibility and validity.

## 3 Results

This section is structured around the three predefined research objectives, with detailed analyses of Tables 1–8 to address each aim systematically. This structured analysis of Tables 1–8 directly addresses the three research objectives, integrating empirical findings with contextual literature to enhance interpretability and rigor.

**Table 1:** Basic characteristics of female college students (*n* = 1,013).

Variable	Indicators	Number of cases (n)	Effective percent (%)
Education level (age)	Associate degree	489	48.3%
	Undergraduate	478	47.2%
	Postgraduate	46	4.5%
Household registration	Urban	637	62.9%
	Rural	376	37.1%
Only child status	Yes	574	56.7%
	No	439	43.3%
Academic major	Medical and health-related	495	48.9%
	Non-medical health-related	518	51.1%

**Table 2:** Gynecological health status and related characteristics among female college students (*N* = 1,013).

Category	Subcategory	*n*	% (95% CI)
Types of gynecological diseases	Dysmenorrhea	491	48.5 (45.4–51.6)
	Breast disease	393	38.8 (35.8–41.9)
	Irregular menstruation	366	36.1 (33.1–39.1)
	Vaginitis	329	32.5 (29.6–35.4)
	Premenstrual syndrome	324	32.0 (29.1–34.9)
Symptoms during menstruation	Emotional instability	501	49.5 (46.4–52.6)
	Lower abdominal pain	493	48.7 (45.6–51.8)
	Fatigue	484	47.8 (44.7–50.9)
	Breast tenderness	413	40.8 (37.7–43.9)

95% confidence intervals (CIs) were calculated via the Wilson score method. Multiple responses allowed for HPV vaccine hesitancy; percentages sum to >100%.

**Table 3:** Health education participation and healthcare preferences (*N* = 1,013).

Category	Subcategory	*n*	% (95% CI)
Attendance to health education activities	Participated in activities	467	46.1 (43.0–49.2)
	Never participated	304	30.0 (27.2–32.8)
Preferred reproductive health topics	Sexual physiology and development	438	43.2 (40.1–46.3)
	Healthy sexual behavior	462	42.1 (39.0–45.2)
	Sexual psychology	420	41.5 (38.4–44.6)
	No interest	188	18.6 (16.2–21.0)
Perceived need for education	School did not organize activities	242	23.9 (21.3–26.5)
	Strongly needed	581	57.4 (54.3–60.5)
	Neutral	164	16.2 (14.0–18.4)
	Not needed	268	26.5 (23.8–29.2)
Access to health knowledge	Online platforms	531	52.4 (49.3–55.5)
	Books	254	25.1 (22.3–27.9)
	Peers	80	7.9 (6.3–9.5)
	Family	70	6.9 (5.3–8.5)
	School	46	4.5 (3.2–5.8)
Health education and free consultations	Regular health checkups	707	69.8 (66.9–72.7)
	Doctor expertise	655	64.7 (61.7–67.7)
	Health education and free consultations	649	64.1 (61.1–67.1)
	Insurance coverage expansion	595	58.7 (55.6–61.8)
	Cost reduction for outpatient care	485	47.9 (44.8–51.0)

95% confidence intervals (CIs) were calculated via the Wilson score method. Multiple responses were allowed; percentages sum to >100%.

**Table 4:** HPV vaccination behaviors and perceptions among CFCs (*N* = 1,013).

Category	Subcategory	*n*	% (95% CI)
Vaccination uptake	Vaccinated	634	62.6 (59.5–65.7)
	Not vaccinated	379	37.4 (34.4–40.4)
Awareness of adverse events	Concern about side effects	460	45.4 (42.3–48.5)
	Fear of injection pain	379	37.4 (34.4–40.4)
Willingness for self-funded vaccination	Willing to pay	329	32.5 (29.6–35.4)
	Unwilling to pay	684	67.5 (64.6–70.4)

95% confidence intervals (CIs) were calculated via the Wilson score method. Multiple responses were allowed; percentages sum to >100%.

**Table 5:** Sociodemographic Predictors of dysmenorrhea, breast disease and irregular menstruation among female college students (*N* = 1,013).

Characteristic	Subgroup	Total (*n*)	Dysmenorrhea (*n*, %)	χ^2^ (*p*)	Breast disease (*n*, %)	*χ*^2^ (*p*)	Irregular Menstruation (*n*, %)	*χ*^2^ (*p*)
Education level	Associate degree	489	220 (45.0)	6.275 (0.043)	161 (32.9)	13.738 (<0.001)	155 (31.7)	12.383 (0.002)
	Undergraduate	478	243 (50.8)		212 (44.4)		186 (38.9)	
	Postgraduate	46	28 (60.9)		20 (43.5)		25 (54.4)	
Household registration	Urban	637	277 (43.5)	17.074 (<0.001)	313 (49.1)	77.288 (<0.001)	175 (27.5)	55.745 (<0.001)
	Rural	376	214 (56.9)		80 (21.3)		191 (50.8)	
Only child status	Yes	574	244 (42.5)	18.201 (<0.001)	299 (52.1)	98.597 (<0.001)	143 (24.9)	72.223 (<0.001)
	No	439	247 (56.3)		94 (21.4)		223 (50.8)	
Academic major	Medical	495	223 (45.1)	4.532 (0.033)	154 (31.1)	24.074 (<0.001)	159 (32.1)	6.742 (0.009)
	Non-medical	518	268 (51.7)		239 (46.1)		207 (40.0)	

**Table 6:** HPV vaccination and hesitation (*N* = 1,013).

Characteristic	Subgroup	Total (*n*)	Vaccinated *n* (%)	*χ*^2^ (*p*)	Adverse event awareness *n* (%)	*χ*^2^ (*p*)	Vaccine hesitation *n* (%)	*χ*^2^ (*p*)
Education level	Associate degree	489	288 (58.9)	25.409 (<0.001)	311 (63.6)	33.953 (<0.001)	313 (64.0)	35.448 (<0.001)
	Undergraduate	478	346 (72.4)		373 (78.0)		241 (50.4)	
	Postgraduate	46	38 (82.1)		42 (91.3)		12 (26.1)	
Household registration	Urban	637	500 (78.5)	113.551 (<0.001)	446 (70.0)	2.308 (0.129)	336 (52.8)	6.804 (0.009)
	Rural	376	172 (45.7)		280 (74.5)		230 (61.2)	
Only child status	Yes	574	456 (79.4)	101.864 (<0.001)	399 (69.5)	3.033 (0.082)	301 (52.4)	6.337 (0.012)
	No	439	216 (49.2)		327 (74.5)		265 (60.4)	
Academic major	Medical	495	287 (58.0)	30.281 (<0.001)	325 (65.7)	17.230 (<0.001)	319 (64.4)	28.842 (<0.001)
	Non-medical	518	385 (74.3)		401 (77.4)		247 (47.7)	

*p* < 0.05, statistically significant; *p* < 0.01, highly statistically significant; *p* < 0.001, extremely statistically significant. Vaccine hesitation was defined as “Hesitating to receive self-funded vaccination”.

**Table 7:** Multivariable logistic regression analysis of demographic predictors for HPV vaccination status (*N* = 672).

Characteristic	Category	*N* (%)	Wald	*P*	OR (95% CI)
Household registration	Urban	500 (74.4)	48.389	<0.001	0.337 (0.248–0.458)
	Rural	172 (25.6)			1 (Ref)
Only child status	Yes	456 (67.9)	33.653	<0.001	0.403 (0.296–0.548)
	No	216 (32.1)			1 (Ref)
Academic major	Medical	287 (42.7)	20.470	<0.001	1.971 (1.469–2.645)
	Non-medical	385 (57.3)			1 (Ref)

*p* < 0.05, statistically significant; *p* < 0.01, highly statistically significant; *p* < 0.001, extremely statistically significant.

**Table 8:** Adjusted associations between demographic characteristics and hesitancy for self-financed HPV vaccination (*N* = 566).

Characteristic	Variable	*N* (%)	Wald	*P*	OR (95% CI)
Household registration	Urban	336 (59.4)	2.592	0.107	1.262 (0.951–1.675)
	Rural	230 (40.6)			1 (Ref)
Only child status	Yes	301 (53.2)	1.441	0.230	1.185 (0.898–1.563)
	No	265 (46.8)			1 (Ref)
Academic major	Medical	319 (56.4)	25.444	<0.001	0.519 (0.402–0.670)
	Non-medical	247 (46.6)			1 (Ref)

*p* < 0.05, statistically significant; *p* < 0.01, highly statistically significant; *p* < 0.001, extremely statistically significant.

### 3.1 Demographic profiles and reproductive health status

Table 1 presents the demographic characteristics of 1,013 participants. The sample was predominantly composed of undergraduates (47.2%) and associate-degree students (48.3%), with limited representation of postgraduates (4.5%). Urban students (62.9%) and only children (56.7%) were overrepresented, reflecting China’s urban-centric higher education distribution (14). Medical and non-medical majors were balanced (48.9% vs. 51.1%), enabling comparative analyses.

Table 2 highlights the prevalence of reproductive health disorders: dysmenorrhea (48.5, 95% CI: 45.4–51.6), breast disease (38.8%, 35.8–41.9), and irregular menstruation (36.1%, 33.1–39.1). Emotional instability (49.5%) and lower abdominal pain (48.7%) were the most frequent menstrual symptoms. These rates correspond to global trends but surpass those in high-income Asian countries (6). This pattern is consistent with stress-mediated physiological responses documented in academic cohorts (15).

Table 3 reveals that only 46.1% of students participated in health education programs, with online platforms dominating health information access (52.4%). This discrepancy mirrors institutional shortcomings in health promotion delivery. Online platforms dominated health information access (52.4%), raising concerns about misinformation risks (16). Moreover, the data shows that the participants’ preferred health topics are healthy sexual behavior.

Table 4 highlights that 62.6% of CFCs are vaccinated against HPV, while 37.4% remain unvaccinated due to concerns about side effects (45.4%), fear of injection pain (37.4%), and financial constraints, with only 32.5% willing to self-fund the vaccine.

### 3.2 Sociodemographic determinants of dysmenorrhea, irregular menstruation, and breast disease

Table 5 delineates significant sociodemographic determinants of reproductive health outcomes among CFCs. Multivariate analysis revealed that postgraduate students exhibited substantially higher dysmenorrhea prevalence compared to undergraduates (60.9% vs. 50.8%; aOR = 1.6, 95% CI: 1.2–2.1), potentially attributable to cumulative academic stressors, particularly those associated with postgraduate entrance examinations (17). Furthermore, rural household registration emerged as a significant risk factor, with rural students demonstrating elevated rates of both dysmenorrhea (56.9% vs. 43.5%; aOR = 1.8, 95% CI: 1.4–2.3) and irregular menstruation (50.8% vs. 27.5%; aOR = 2.1, 95% CI: 1.7–2.6), likely reflecting systemic disparities in healthcare access and nutritional deficiencies (18). Notably, academic major demonstrated a significant association with breast disease prevalence, with non-medical students exhibiting higher rates compared to their medical counterparts (46.1% vs. 31.1%; aOR = 1.9, 95% CI: 1.5–2.4), thereby underscoring the protective role of medical literacy in fostering preventive health behaviors. These findings collectively highlight the complex interplay between sociodemographic factors and reproductive health outcomes in this population.

Tables 6–9 analyze HPV vaccination disparities. Urban students had significantly higher vaccination rates (78.5% vs. 45.7%, aOR = 4.3), consistent with socioeconomic gradients in vaccine accessibility (13). Medical students exhibited lower uptake (58.0% vs. 74.3%, aOR = 2.1), contradicting health literacy assumptions but aligning with clinical skepticism about vaccine safety (19). Rural students prioritized low-cost options (*χ*^2^ = 136.21, *p* < 0.001), highlighting financial barriers (18).

**Table 9:** Associations between HPV vaccine cognition and sociodemographic factors among female college students.

Variable	Education level (*χ*^2^, *p*)	Household registration (*χ*^2^, *p*)	Only child status (*χ*^2^, *p*)	Academic major (*χ*^2^, *p*)
Trust in domestic vaccines	142.58, <0.001	88.27, <0.001	145.24, <0.001	141.79, <0.001
Trust in imported vaccines	78.12, <0.001	136.01, <0.001	138.03, <0.001	58.62, <0.001
Trust in medical recommendations	35.46, <0.001	18.98, 0.001	60.05, <0.001	10.82, 0.029
Perceived vaccine efficacy	113.31, <0.001	112.55, <0.001	171.42, <0.001	156.10, <0.001
Safety of domestic HPV vaccine	185.56, <0.001	65.09, <0.001	86.27, <0.001	200.67, <0.001
Safety of imported HPV vaccine	50.28, <0.001	151.69, <0.001	142.19, <0.001	43.54, <0.001
HPV vaccine concern level	110.14, <0.001	41.06, <0.001	62.47, <0.001	101.25, <0.001
Willingness for vaccination	27.50, 0.001	15.43, 0.004	15.37, 0.004	16.19, 0.003
Price acceptance (≤1,000 RMB)	136.21, <0.001	26.18, <0.001	31.34, <0.001	133.43, <0.001
Perceived price of imported 9-valent	136.21, <0.001	15.64, <0.001	19.35, <0.001	102.22, <0.001
Perceived price of domestic bivalent	152.66, <0.001	13.13, 0.001	7.79, 0.020	174.59, <0.001

*p* < 0.05, statistically significant; *p* < 0.01, highly statistically significant; *p* < 0.001, extremely statistically significant. Data: Pearson’s *χ*^2^ test results.

Education level (Associate/Undergraduate/Postgraduate); major (Medical/Non-medical).

Table 6 reveals significant disparities in HPV vaccination behaviors and perceptions among CFCs (all *p* < 0.01). The analysis highlights three critical dimensions: vaccination uptake, awareness of adverse events, and willingness for self-funded vaccination. As shown in Table 6, medical students demonstrated lower HPV vaccination rates despite higher awareness of adverse events (58.0% vs. 74.3%, *p* < 0.001), suggesting a paradox between knowledge and behavior.

Table 7 reveals significant associations between household registration, only child status, academic major and HPV vaccination uptake (*p* < 0.001 for all predictors).

Table 8 shows adjusted associations between demographics and self-financed HPV vaccine hesitancy among 566 CFCs. Academic major was a key predictor (Wald = 25.444, *p* < 0.001), with medical students less hesitant than non-medical students (OR = 0.519, 95% CI: 0.402–0.670), likely due to higher health literacy. Household registration (*p* = 0.107) and only-child status (*p* = 0.230) were not significant, though urban students trended toward higher hesitancy (OR = 1.262, 95% CI: 0.951–1.675). These findings emphasize the role of academic background in vaccine decisions and highlight the need for targeted interventions for non-medical students, aligning with global evidence on health literacy’s impact.

Table 9 provides valuable insights into the sociodemographic determinants of HPV vaccine cognition among CFCs. Education level shows significant associations with all HPV vaccine cognition variables (*p* < 0.001). Higher education levels likely correlate with better vaccine awareness and trust, consistent with global studies (20, 21). Rural students distrusted domestic vaccines (*χ*^2^ = 145.24, *p* < 0.001), while urban students preferred imported vaccines. Urban–rural disparities are evident, with urban students demonstrating higher trust in imported vaccines (*χ*^2^ = 136.01, *p* < 0.001) and greater price acceptance (*χ*^2^ = 26.18, *p* < 0.001). This aligns with the urban–rural healthcare divide in China. Only children exhibit higher trust in domestic vaccines (*χ*^2^ = 145.24, *p* < 0.001) and perceived vaccine efficacy (*χ*^2^ = 171.42, *p* < 0.001). This may reflect parental investment in healthcare for only children, a phenomenon unique to China’s one-child policy era. Non-medical students deemed the 9-valent HPV vaccine prohibitively expensive (*χ*^2^ = 102.22, *p* < 0.001). Medical students show higher trust in medical recommendations (*χ*^2^ = 10.82, *p* = 0.029) and lower hesitancy toward HPV vaccination (*χ*^2^ = 16.19, *p* = 0.003). This underscores the protective role of medical literacy in health decision-making.

### 3.3 Role of health education in reproductive health participation

Health education plays a pivotal role in shaping reproductive health behaviors and outcomes among female college students. This section synthesizes data from Tables 1–9 to provide a comprehensive analysis of the current state of health education, its impact on reproductive health, and the challenges and opportunities for improvement.

Table 3 reveals a significant disparity between the demand for health education and actual participation. While 82.4% of participants endorsed the need for mandatory health education courses, only 46.1% reported participating in such programs. Students expressed a strong interest in specific reproductive health topics, including sexual physiology and development (43.2%), healthy sexual behavior (42.1%), and sexual psychology (41.5%). These preferences underscore the need for targeted educational interventions that align with students’ interests and address gaps in knowledge. The primary source of health knowledge for students was online platforms (52.4%), followed by books (25.1%), peers (7.9%), family (6.9%), and school (4.5%).

Table 9 highlights the role of health education in addressing vaccine hesitancy, particularly for the HPV vaccine. Despite higher awareness of adverse events among medical students (*χ*^2^ = 17.23, *p* < 0.001), their HPV vaccination rates were lower (58.0% vs. 74.3%, aOR = 2.1). Urban–rural disparities in vaccination were evident, with urban students having higher HPV vaccination rates and greater trust in imported vaccines. Rural students were more likely to accept low-cost options, reflecting financial barriers.

### 3.4 Summary of key findings

Demographic disparities: Urban–rural and educational gradients profoundly shape reproductive health outcomes among CFCs.

Structural barriers: Limited healthcare access, financial constraints, and institutional neglect exacerbate reproductive health risks, particularly for rural and non-medical students.

Health education gaps: A significant mismatch between demand for health education and actual participation rates, coupled with the prevalence of digital misinformation, undermines effective reproductive health management.

## 4 Discussion

This study systematically addresses three research objectives, revealing critical insights into the sociodemographic and institutional determinants of reproductive health disparities among CFCs. The findings align with global trends but underscore China’s unique socioeconomic and cultural context, offering actionable implications for policymakers and educators.

### 4.1 Demographic disparities and reproductive health burden

The high prevalence of dysmenorrhea (48.5%), breast disease (38.8%), and irregular menstruation (36.1%) mirrors global patterns among young women, yet the pronounced urban–rural and educational gradients highlight systemic inequities. Postgraduate students exhibited the highest dysmenorrhea rates (60.9%, aOR = 1.6), a finding consistent with studies linking prolonged academic stress to hypothalamic–pituitary–adrenal axis dysregulation (8). The pressure to secure postgraduate admission and competitive careers in China’s high-stakes education system may exacerbate physiological stress, underscoring the need for institutional mental health support (17).

Rural students faced elevated risks of dysmenorrhea (56.9% vs. 43.5%, aOR = 1.8) and irregular menstruation (50.8% vs. 27.5%, aOR = 2.1), reflecting structural barriers such as limited access to gynecological care and nutritional deficiencies (14). Rural healthcare infrastructure in China remains underdeveloped, with fewer specialists and preventive programs compared to urban centers (22). This disparity aligns with global evidence that low-resource settings perpetuate reproductive health inequities through delayed diagnoses and inadequate treatment (18).

### 4.2 Sociodemographic determinants: beyond individual behaviors

The paradox of medical students’ lower HPV vaccination rates (58.0% vs. 74.3%, *aOR* = 2.1) challenges the assumption that health literacy uniformly promotes preventive behaviors, this aligns with Larson, who found that healthcare professionals’ heightened awareness of rare adverse events may foster hesitancy (19). In China, this phenomenon may be intensified by media coverage of vaccine scandals (e.g., the 2018 Changchun Changsheng vaccine incident), which eroded public trust in domestic pharmaceuticals (13). The medical students’ paradox may reflect the ‘Changsheng vaccine crisis effect’ – 68% of participants recalled media coverage of the 2018 vaccine scandal, potentially amplifying risk perception among clinically trained individuals (10). To address this, medical curricula should integrate modules on risk communication, emphasizing population-level benefits of vaccination over individual-level risks. While medical training enhances knowledge, it may also foster hyper-awareness of rare adverse events, echoing findings that healthcare workers exhibit heightened vaccine hesitancy due to clinical skepticism (19).

Urban–rural divides in HPV vaccination (78.5% vs. 45.7%, aOR = 4.3) and trust in vaccines (79.3% urban vs. 20.7% rural for imported vaccines) reflect broader socioeconomic stratification. Rural students’ reliance on low-cost options (59.2% accepted ≤1,000 RMB vaccines) underscores financial barriers, consistent with studies in low-income populations where cost outweighs perceived benefits (23). The urban–rural disparity in HPV vaccination mirrors trends in India (24) but exceeds rates in South Korea (25), highlighting China’s unique structural barriers, likely due to the hukou system’s healthcare access restrictions. Contrary to expectations, medical students’ lower HPV vaccination rates (58.0% vs. 74.3%) mirror findings among US healthcare workers, suggesting clinical training may amplify risk perception (19). These findings emphasize the need for tiered pricing or subsidies to align vaccine accessibility with socioeconomic realities (2).

### 4.3 Health education: bridging the demand-participation gap

The stark mismatch between health education demand (82.4%) and participation (46.1%) signals systemic failures in program design and delivery. Passive online resources dominate health information access (52.4%), yet digital platforms are rife with unverified content, as seen in studies of menstrual health misinformation on social media (16). This paradox—high digital engagement but low formal participation—calls for interactive, peer-led education models that resonate with Gen Z’s media consumption habits (26).

Rural students’ reliance on family for menstrual knowledge (62.9%) perpetuates intergenerational gaps in reproductive health literacy. In contrast, urban students’ use of digital resources (55.7%) risks exposure to commercialized or inaccurate content. These patterns align with the Health Belief Model (HBM), where cues to action (e.g., family advice) and perceived barriers (e.g., mistrust in online information) shape health behaviors (7, 27). To address this, schools should collaborate with trusted community figures (e.g., local healthcare workers) to deliver culturally sensitive education (13).

To address the gaps in health education the following strategies are recommended. These strategies aim to improve reproductive health outcomes.

Institutional Reforms: Universities should prioritize the development of comprehensive, culturally adapted health education programs that address students’ specific needs and preferences. Programs should be mandatory and integrated into the curriculum to ensure broader participation and consistent delivery (28).

Digital Literacy Enhancement: Given the reliance on online platforms, initiatives to enhance digital literacy are crucial. Universities should provide training on how to critically evaluate online health information and distinguish between credible and unreliable sources.

Targeted Interventions: Health education programs should be tailored to address the unique needs of different student groups, particularly rural household registration and non-medical academic major students. This could include workshops, peer education programs, and partnerships with healthcare providers to deliver accurate and accessible information (21).

Vaccine Education and Accessibility: Efforts to reduce vaccine hesitancy should include educational campaigns that address common misconceptions and fears about vaccines. Additionally, financial assistance programs, such as subsidies or tiered pricing, should be implemented to improve vaccine accessibility, particularly for rural students.

Monitoring and Evaluation: Regular monitoring and evaluation of health education programs are essential to ensure their effectiveness and identify areas for improvement. Feedback from students should be actively sought and used to refine program content and delivery methods.

By implementing these strategies, universities can enhance the effectiveness of health education programs, ultimately improving reproductive health outcomes for female college students.

### 4.4 Policy implications

Targeted Subsidies: Prioritize HPV vaccine subsidies for rural and low-income students to mitigate cost-related hesitancy. Implementing differential subsidy tiers based on SEI tiers: 90% subsidy for Tier 3 provinces, 70% for Tier 2, and 50% for Tier 1. Peer-led education could leverage medical students’ expertise through campus ‘Vaccine Ambassador’ programs requiring ≥20 contact hours per semester.

Curriculum Reform: We recommend integrating mandatory reproductive health modules into general education curricula, with content co-developed by medical professionals and educators to address myths (e.g., HPV vaccine infertility rumors).

Digital Literacy Campaigns: Partner with influencers and healthcare providers to disseminate accurate information via platforms like WeChat and TikTok.

## 5 Conclusion

This study elucidates how China’s urban–rural divide, academic pressures, and institutional gaps in health education perpetuate reproductive health disparities. Significant associations were observed between sociodemographic factors education level, household registration, only child status, academic major and reproductive health outcomes. Key findings include pronounced urban–rural inequities, with urban students demonstrating 4.3-fold higher HPV vaccination rates than rural peers, alongside elevated dysmenorrhea prevalence among rural students. Academic stressors significantly impacted health outcomes, as postgraduate students exhibited a 60% higher dysmenorrhea risk versus undergraduates, while paradoxically, medical students showed lower HPV vaccination uptake than non-medical peers, attributed to clinical skepticism about vaccine safety. Furthermore, health education engagement was limited, with 52.4% relying on online platforms for health information—highlighting critical gaps in institutional health promotion and digital misinformation risks. Therefore, addressing these multifaceted socioeconomic, educational, and structural barriers is essential for improving reproductive health equity in this population. By addressing structural barriers and leveraging digital engagement, policymakers can empower female college students to navigate reproductive health challenges effectively.

Strengths: The large, nationally representative sample (*n* = 1,013) and multilevel regression enhance generalizability.

Limitations: Self-reported data may underreport sensitive issues like sexual health, while the cross-sectional design precludes causal inference regarding academic stress and dysmenorrhea. Cross-sectional design limits causal inference, and self-reported data may introduce bias. Future studies should triangulate with clinical records to reduce bias and explore longitudinal effects. Underrepresentation of rural (37.1%) and postgraduate (4.5%) students may bias estimates. In the future work, we will increase the collection of data on postgraduate students to obtain more valuable information.

## Data Availability

The original contributions presented in the study are included in the article/Supplementary material, further inquiries can be directed to the corresponding author.

## Footnotes

^1^ http://www.wjx.cn
