# Supplementary material for: Factors associated with reproductive health and health education participation among female college students in China
Source: Front Public Health. 2025 Sep 1;13:1627669. doi: 10.3389/fpubh.2025.1627669 (PMC12433993; doi:10.3389/fpubh.2025.1627669)
Supplement: Supplementary file 1 [file Data_Sheet_1.docx]

**Questionnaire on Reproductive Health and Health Education Participation Among Female College Students in China**

Dear Participant,

Thank you for participating in this important study on reproductive health and health education among female college students in China. Your responses will help us understand the current status and identify areas for improvement in reproductive health education and services. This survey is anonymous, and all data will be kept confidential. The questionnaire consists of three parts: basic information, reproductive health status, and HPV vaccine awareness. Please answer all questions honestly.

For any questions, please contact:

Dr. Wenming Cao

Email: caowenming1983@126.com

Tel: 86-13686368551

**Part One: Your basic information**

**1. Your age: ____ years old.**

**2. Current degree program**

① Associate degree ② Undergraduate ③ Postgraduate

**3. Household registration (hukou)**

① Urban ② Rural

**4. Are you an only-one child?**

① Yes ② No

**5. Academic major:**

① Medical or health-related ② Non-medical

**6. Primary source of income:**

①Parents ②Relatives/friends ③Part-time work ④Student loans

⑤Scholarships ⑥Other (specify): ____

**Part Two: Reproductive Health Status and Knowledge**

**7. Your age of initial menstruation is at ____ years old.**

**8. How did you know about** **menstruation?**

① Parents and relatives ② Classmates and friends ③ Medical personnel

④ School teachers ⑤ Books, internet, videos, TV ⑥ Other sources

**9. Does your physical condition during menstruation affect your study and life?**

① Serious impact ② Modest impact ③ Little impact

**10. Can you daily make your intimate parts washed?**

① Every day ② Nearly daily ③ Sometimes ④ Occasional ⑤ Never

**11. How do you wipe after bowel movement?**

① From front to back ② From back to front

**12. What kind of underwear do you usually wear?**

① Cotton (e.g. cotton) ② Chemical fibre (e.g. nylon) ③ Mixed wearing

**13. Are you subject to gynaecological discomfort recently?**

① Yes ② No

**14. Were you subject to an unwanted pregnancy?**

① Yes ② No

**15. Do you know the safe period, external spermulation, tools and drugs and other contraceptive methods?**

① Familiar ② Generally know ③ Know a little ④ Unknown

**16. Have you ever joined in any activities on reproductive health conducted by your university?**

① Have participated ② Have not participated ③ No related activities

**17. Do you need to receive reproductive health education?**

① Necessary ② Do not care ③ Unnecessary

**18. Is it necessary to establish reproductive health courses at universities?**

① Necessary ② Unnecessary

**19. How do you access to knowledge on reproductive health?**

① Science books ② Internet ③ Classmates and friends ④ Radio and TV

⑤ Family education ⑥ School education

**20. What do you care most when choosing a hospital?**

① Doctor’s professional competence ② Good environment and service attitude

③ Low medical cost ④ Advanced medical facilities ⑤ Privacy

⑥ Acquaintances

**21. What measures should be made to improve the medical insurance for tertiary female students’ reproductive health? (Multiple choice)**

① Provide knowledge dissemination and free-of-charge counselling

② Provide regular medical check-ups

③ Increase the reimbursement rate of medical expenses on gynaecological outpatient

④ Expand the coverage of maternity insurance

**22. What symptoms do you experience pre-post menstruation? (Multiple choice)**

① Pain on lower abdominal ② Pain on waist ③ Breast distending pain

④ Headache ⑤ Swollen limbs ⑥ Fatigue

⑦ Impatience, depression, anxiety, sadness, over-sensitivity, suspicion, emotional instability, etc.

**23. What symptoms have you experienced recently? (Multiple choice)**

① Abnormal leucorrhoea ② Irregular vaginal bleeding ③ Itchy intimate parts

④ Pain in the lower back and abdomen ⑤ Breast pain ⑥ No symptoms

**24. What kind of gynaecological diseases have you been faced? (Multiple choice)**

① Dysmenorrhoea ② Irregular menstruation ③ Amenorrhoea

④ Premenstrual syndrome ⑤ Vaginitis ⑥ Cervicitis

⑦ Pelvic inflammatory disease ⑧ Ovarian disease ⑨ Breast Disease ⑩ None

**25. What do you want to know most about reproductive health? (Multiple choice)**

① Healthy sexual behaviour

② Sexually transmitted diseases and gynaecological disease prevention

③ Contraceptive knowledge

④ Anatomy and basic functions of male and female genitals

⑤ Sexual dysfuncti

⑥ Infertility Sexual dysfunction

⑦ Psychosexual knowledge

⑧ Knowledge of sexual morality and ethics

⑨ Knowledge of sexual physiology and development

⑩ Don’t want to know anything

**Part Three:Questionnaire on HPV Vaccine Awareness Survey**

**26. Have you ever received the HPV vaccine?**

① Yes ② No

**27. How much trust do you have in domestic vaccine manufacturers?**

①Very trusting ②Relatively trusting ③Average

④Not very trusting ⑤Very distrustful

**28. How much trust do you have in foreign vaccine manufacturers?**

① Very trusting ② Relatively trusting ③ Average

④ Not very trusting ⑤ Very distrustful

**29. How much trust do you have in the vaccinations recommended by medical staff?**

①Very trusting ②Relatively trusting ③Average

④ Not very trusting ⑤Very distrustful

**30. Have you ever read the reports in the media about the adverse vaccine incidents of manufacturing enterprises?**

① Yes ② No

**31. Do you think it is necessary to get vaccinated against HPV?**

① Necessary ② Unnecessary

**32.How concerned are you about the HPV vaccine?**

① Very concerned ② Relatively concerned ③ Average

④ Not very concerned ⑤ Very not concerned

**33.Which of the following channels do you most often obtain knowledge about HPV vaccines from?**

① Internet ② Television ③ Radio ④ Books ⑤ Others

**34.How much trust do you have in the effectiveness of vaccines in preventing cervical cancer?**

①Very trusting ②Relatively trusting ③ verage

④Not very trusting ⑤Very distrustful

**35.How much trust do you have in the safety of the current domestic bivalent HPV vaccine?**

①Very trusting ②Relatively trusting ③Average

④ Not very trusting ⑤ Very distrustful

**36.How much trust do you have in the safety of the currently imported HPV vaccines?**

①Very trusting ②Relatively trusting ③Average

④ Not very trusting ⑤ Very distrustful

**37.The imported 9-valent HPV vaccine is approximately 3,999 yuan. What's your opinion on the price of this vaccine?**

① Moderate price ② High price ③ Low price

**38.At present, the domestic bivalent HPV vaccine is a self-paid vaccine, with a price close to 1,000 yuan. What's your opinion on the price of this vaccine?**

① Moderate price ② High price ③ Low price

**39.What is the price of the HPV vaccine that you can accept?**

① Under 1,000 RMB ② 1,000-1,999 RMB ③ 2,000-2,999 RMB

④ 3,000-3,999 RMB ⑤ 4,000 RMB and above ⑥Uncertain

**40. What is your willingness to get the HPV vaccine?**

①Very willing ②Relatively willing ③Uncertain

④Not very willing ⑤ Very unwilling 

**41.What are the reasons why you are reluctant to get the HPV vaccine? (Multiple choice)**

① Not familiar with the vaccine

② Thinking that the chance of getting cervical cancer is very small

③ Not believing that the vaccine can prevent cervical cancer

④ Fearing that the vaccine has side effects 

⑤ The price of the vaccine is too high

⑥ The process of making an appointment for vaccination is too complicated

⑦ Relatives and friends do not support getting vaccinated

⑧ Fear that the vaccination process will be too painful

⑨ The vaccine is not widely available

⑩ Others

**42. Are you hesitant about getting the HPV vaccine at your own expense at present?**

① There was hesitation ② There was no hesitation

Thank You!

Your participation is invaluable.

For further inquiries, please contact the research team. Best wishes!

**女大学生生殖健康状况的调查问卷**

亲爱的同学：

您好!为了能更好地了解女大学生的生殖健康状况，完善女大学生生殖健康教育，保障女大学生生殖健康权益，我们组织了本次调查。真诚地希望大家能够理解，并积极配合，您所提供的每一项信息对于我们都是很重要的。

本调查无记名，信息严格保密。谢谢您的配合与支持!

填写说明：除特殊说明外，下列各题均为单选题，请在您选择的序号上打“√”。如果没有合意的选项，请您在“____”上填写文字。

联系人：曹文明

联系电话：13686368551

**一、基本情况**

**1、您的年龄**：____周岁。

**2、您现在就读的年级?**

①专科 ②本科 ③硕士及以上

**3、您的原户籍所在地**：

①城镇 ②农村

**4、您是独生子女吗?**

①是 ②不是

**5、您所学专业是：**

①医学卫生相关专业 ②非医学卫生相关专业

1. **您最主要的经济来源是：**

①父母提供 ②亲戚朋友资助 ③兼职收入

④助学贷款 ⑤奖学金 ⑥其它_______(请说明)

**二、生殖健康状况及健康教育知晓情况**

7、**您的月经初潮年龄是____**周岁

**8、您的月经知晓途径是?**

①父母及家人 ②同学及朋友 ③医务人员 ④学校老师

⑤图书、网络、视频、电视 ⑥其它

**9、月经期的身体状况会对学习与生活产生影响吗?**

①严重影响 ②影响 ③不影响

**10、您能保证每天清洗私密处吗?**

①每天都会 ②基本上都会 ③有时会 ④偶尔会 ⑤不会

1. **您大便后的擦拭方法是?**

①从前到后 ②从后到前

1. **您平日习惯穿着什么材质的内裤?**

①棉质（如纯棉） ②化纤（如尼龙） ③混合穿

1. **您近期是否有妇科方面的不适?**

①是 ②否

**14、您是否发生过意外怀孕?**

①是 ②否

**15、您是否了解安全期、体外排精、工具及药物等避孕方法?**

①非常了解 ②一般了解 ③不大了解 ④一点不懂

**16、您是否参加过学校开展的生殖健康教育活动?**

①参加过 ②没有参加过 ③学校没有开展过

**17、您认为自己需要接受生殖健康教育吗?**

①需要 ②无所谓 ③不需要

**18、你觉得学校有必要开展生殖健康方面的课程吗?**

①有必要 ②没必要

**19、您现在所了解的有关生殖健康的知识，主要是通过以下哪个渠道?**

①科普读物 ②网络 ③同学及朋友 ④广播电视 ⑤家庭教育 ⑥学校教育

**20、您选择医院就诊时，最看重什么?**

①对医生技术水平的信任 ②较好的就医环境和服务态度 ③低廉的医疗费用

④先进的医疗设备 ⑤保密个人隐私 ⑥熟人介绍

**21、你觉得大学生医疗保障在女大学生健康方面还应做出哪些改进?(可多选)**

①提供生殖健康知识宣传及免费咨询 ②提供定期健康体检

③提高妇科门诊医疗费的报销比例 ④扩大生育保险的保障范围

**22、每次月经来潮前及经期，你会出现以下哪些症状?(可多选)**

①下腹坠痛 ②腰痛 ③乳房胀痛 ④头痛 ⑤四肢肿胀

⑥疲劳乏力 ⑦急躁、抑郁、焦虑、忧伤、过度敏感、猜疑、情绪不稳等

**23、您近期出现过以下哪些症状?(可多选)**

①白带异常 ②不规则阴道流血 ③私处瘙痒 ④腰腹部疼痛

⑤乳房胀痛 ⑦无任何症状

**24、您曾经患有哪种妇科疾病?(可多选)**

①痛经 ②月经不调 ③闭经 ④经前期综合症 ⑤阴道炎

⑥宫颈炎 ⑦盆腔炎 ⑧卵巢疾病 ⑨乳腺疾病 ⑩无任何症状

**25、您最想了解哪些方面的生殖健康知识?(可多选)**

①健康的性行为 ②性病及妇科病预防知识 ③避孕知识

④男女生殖器解剖及基本功能 ⑤性功能障碍 ⑥不孕不育的治疗

⑦性心理知识 ⑧性道德伦理 ⑨性生理及发育知识 ⑩不想了解任何知识

**三、HPV疫苗认知调查问卷**

**26、您是否接种过HPV疫苗？**

①是 ②否

**27、您对国内疫苗生产企业的信任程度?**

①非常信任 ②比较信任 ③一般 ④不太信任 ⑤非常不信任

**28、您对国外疫苗生产企业的信任程度?**

①非常信任 ②比较信任 ③一般 ④不太信任 ⑤非常不信任

**29、您对医务人员推荐接种疫苗的信任程度?**

①非常信任 ②比较信任 ③一般 ④不太信任 ⑤非常不信任

**30、您是否看过媒体中对生产企业不良疫苗事件的报道?**

①是 ②否

**31、您认为接种HPV疫苗是否有必要?**

①有必要 ②没有必要

**32、您对HPV疫苗的关注程度为?**

①非常关注 ②比较关注 ③一般 ④不太关注 ⑤非常不关注

**33、您最常从以下哪种渠道获取HPV疫苗的知识?**

①网络 ②电视 ③广播 ④书籍 ⑤其他

**34、您对疫苗预防宫颈癌效果的信任程度为?**

①非常信任 ②比较信任 ③一般 ④不太信任 ⑤非常不信任

**35、您对目前国产的二价HPV疫苗安全性的信任程度？**

①非常信任 ②比较信任 ③一般 ④不太信任 ⑤非常不信任

**36、您对目前进口的HPV疫苗安全性的信任程度？**

①非常信任 ②比较信任 ③一般 ④不太信任 ⑤非常不信任

**37、进口九价HPV疫苗约为3999元。您对该疫苗价格的看法是?**

①价格适中 ② 价格偏高 ③价格偏低

**38、目前国产二价HPV疫苗属于自费疫苗，价格接近1000元。您对该疫苗价格的看法是？**

①价格适中 ② 价格偏高 ③价格偏低

**39、您能接受的HPV疫苗价格为？**

①1000元以下 ②1000-1999元 ③2000-2999元 ④3000-3999元

⑤4000元及以上 F.不确定

**40、您接种HPV疫苗的意愿为？**

①非常愿意 ②比较愿意③不确定④不太愿意 ⑤非常不愿意

**41、您不愿意接种HPV疫苗的原因有以下几项？（多选题）**

①不了解该疫苗 ②认为自己得宫颈癌的几率很小

③不相信疫苗能够预防宫颈癌 ④害怕该疫苗有副作用

⑤疫苗价格太高 ⑥预约接种程序太麻烦⑦亲朋好友不支持接种疫苗 ⑧怕接种过程太疼 ⑨疫苗不够普及 ⑩其他

**42、您对目前自费接种HPN疫苗是否会有犹豫?**

①有犹豫 ②没有犹豫

感谢您的支持和帮助，祝您身体健康，学业顺利，万事如意!
